# C2 Fracture Subtypes, Incidence, and Treatment Allocation Change with Age: A Retrospective Cohort Study of 233 Consecutive Cases

**DOI:** 10.1155/2017/8321680

**Published:** 2017-01-15

**Authors:** Anna-Lena Robinson, Anders Möller, Yohan Robinson, Claes Olerud

**Affiliations:** ^1^Department of Surgical Sciences, Uppsala University Hospital, Uppsala, Sweden; ^2^Stockholm Spine Center, Stockholm, Sweden; ^3^Department of Orthopaedics, Skåne University Hospital, Malmö, Sweden

## Abstract

The currently available data on the distribution of C2 fracture subtypes is sparse. This study was designed to identify the proportions of the second cervical vertebra (C2) fracture subtypes and to present age and gender specific incidences of subgroups. A dataset of all patients treated between 2002 and 2014 for C2 fractures was extracted from the regional hospital information system. C2 fractures were classified into odontoid fractures types 1, 2, and 3, Hangman's fractures types 1, 2, and 3, and atypical C2 fractures. 233 patients (female 51%, age 72 ± 19 years) were treated for a C2 fracture. Odontoid fractures were found in 183 patients, of which 2 were type 1, 127 type 2, and 54 type 3, while 26 of C2 fractures were Hangman's fractures and 24 were atypical C2 fractures. In the geriatric subgroup 89% of all C2 fractures were odontoid, of which 71% were type 2 and 29% type 3. There was an increasing incidence of odontoid fractures types 2 and 3 from 2002 to 2014. 40% of C2 fractures were treated surgically. This study presents reliable subset proportions of C2 fractures in a prospectively collected regional cohort. Knowledge of these proportions facilitates future epidemiological studies of C2 fractures.

## 1. Introduction

Fractures of the second cervical vertebra (C2) are the most common cervical spinal injury among elderly [1–4]. The most common C2 fracture—the odontoid fracture—has a biphasic age distribution with peaks both at 20–30 and at 70–80 years of age [3, 5]. This finding is not uncommon among spinal fractures [6, 7]. Younger patients are more susceptible to high-energy trauma-related injuries, while elderly sustain bone density-related injuries [1, 2]. Due to a stiffer lower cervical spine, the aged upper cervical spine is susceptible to bony and ligamentous injuries, which—together with reduced bone density—explains the disproportionally high proportion of upper cervical injuries in elderly [4, 8].

C2 fractures can be subdivided into odontoid fractures, Hangman's fractures, and atypical fractures [9]. Since C2 fracture types are associated with different fracture mechanisms, the distribution of various fracture types differ between younger and older patients [10].

Improved motor vehicle safety and protective gear have reduced the incidence of spinal injuries in the younger population [2]. Simultaneously, demographic changes have led to a dramatic increase of the elderly population in Europe [11]. Consequently, a change in distribution and incidence of the various fracture types towards typical osteoporotic fractures can be anticipated [12]. The demographic changes may also have affected the incidence of C2 fractures.

There is no population-based data available on the C2 fracture subgroup distribution with regard to patient age. This study aims at establishing the distribution of C2 fracture subtypes, as well as the age and gender specific annual incidence of various C2 fracture subgroups, and to present the current treatment strategies related to these fractures in a well-defined regional cohort.

## 2. Patients and Methods

In Sweden all patient contacts within the public healthcare system are registered prospectively in a national patient registry with unique personal identification number and diagnosis codes using the 10th version of the International Classification of Diagnosis (ICD-10) [13]. All patients treated at two university hospitals in Sweden (Uppsala and Malmö) between January 2002 and December 2014 with the diagnosis of a C2 fracture (ICD-10: S12.1) were extracted from the regional hospital information system. Patients treated for C2 fractures at the university hospitals, but with a home address, at the time of injury, outside of the county were excluded. Since both hospitals are the only spinal fracture care providers in their respective region, it can be assumed that the coverage is comprehensive.

The patients' radiographs were reviewed retrospectively and C2 fractures were classified independently by two experienced spine surgeons. Odontoid fractures were classified, according to Anderson and D'Alonzo [14], into type 1, type 2, and type 3 referred to as O1, O2, and O3 in the text, tables, and figures, using the modified classification system by Grauer et al. [15] to separate the shallow O3 from O2. Hangman's fractures were classified according to Effendi et al. [16] into type 1, type 2, and type 3 referred to as H1, H2, and H3. Patients that did not fulfil the criteria of any of these classification systems were classified as atypical C2 fractures—referred to as A.

The primary treatment strategy for each patient, surgical or nonsurgical, was retrieved from the hospital records.

The patients were subdivided according to age classes, <70 and ≥70 years. To establish the population-at-risk in the various age and gender groups, population data was retrieved from Statistics Sweden, an administrative agency providing statistics to government and researchers.

### 2.1. Statistics

For statistical analysis SPSS 22.0 (IBM, USA) was used.

The descriptive and the specific annual incidence and treatment strategy were calculated for each age and gender subgroup. Mean values were presented with mean ± standard deviation in the following text. Trends were analysed with linear regression and presented with correlation coefficient *r*. Effects of age group and gender on treatment allocation and C2 fracture distribution were tested with Chi-square test. A *p* value < 0.05 was considered as statistically significant.

## 3. Ethics

The study was approved by the Regional Ethics Committee of Uppsala (number 2010/131/1).

## 4. Results

### 4.1. Fracture Classification and Distribution

During the 13 years of this study the population in Uppsala County and Malmö municipal increased from 432,293 in 2002 to 519,152 in 2014, with a mean age of 39.6 years in 2002 and 39.4 years in 2014. The proportion of elderly ≥70 years decreased from 15.7% in 2002 to 14.9% in 2014.

Two-hundred and thirty-three patients (118 women) with a mean age of 72 ± 19 years (range 17–97) were treated for a C2 fracture. There were 183 odontoid fractures, 26 Hangman's fractures, and 24 atypical C2 fractures. The mean age for odontoid fractures was 76 ± 17 years, for Hangman's fractures 60 ± 20 years, and for atypical fractures 61 ± 24 years of age.

**Figure 1:**
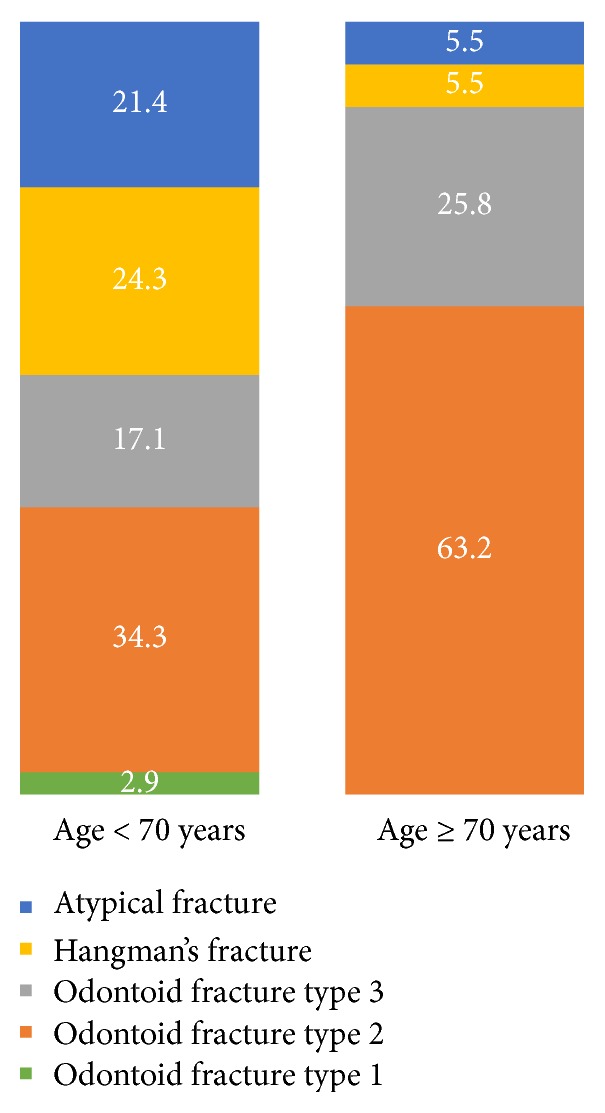
Distribution of C2 fractures divided into patients < 70 years and ≥70 years of age (%).

**Figure 2:**
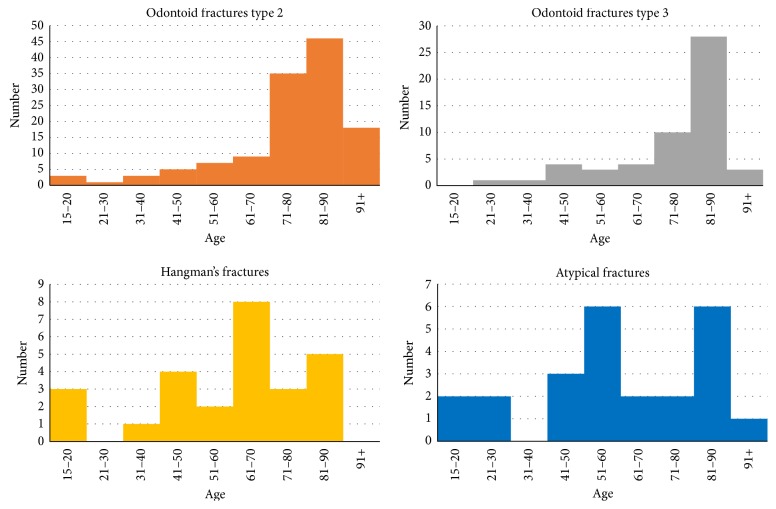
Age distribution of C2 fracture subtypes.

The age group < 70 consisted of 70 patients (20 women), with a mean age of 48 ± 15 years. Thirty-eight of the C2 fractures were odontoid, 17 were Hangman's, and 15 were atypical. The age group ≥ 70 consisted of 163 patients (98 women), with a mean age of 83 ± 7 years. One-hundred and forty-five of the fractures were odontoid, 9 were Hangman's, and 9 were atypical. The age distribution of the various C2 fracture subgroups is presented in Figure 1. In the age group ≥ 70 years, O2 and O3 fractures dominated, whereas there was a more even distribution among the various fracture types in the younger age group. There was a difference between the two age groups with regard to fracture classification and gender (*p* < 0.05). The proportion of O2 decreased compared to O3 from 2002 to 2014 in the age group ≥ 70 years (*p* < 0.05). A more specific age distribution of C2 fractures is shown in Figure 2.

### 4.2. Incidence

**Figure 3:**
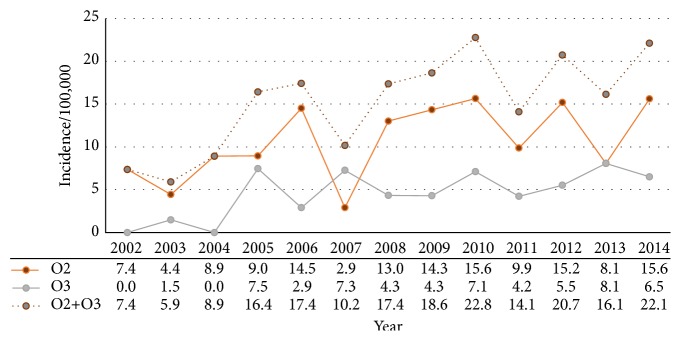
Annual incidence of odontoid fractures (all types) in patients ≥ 70 years (dotted), divided into subgroups of odontoid fracture type 2 (orange) and type 3 (grey) during the years 2002 to 2014.

The incidence of C2 fractures during the entire study period from 2002 to 2014 was 3.8 per 100,000 person-years in the population of Uppsala County and Malmö municipal. In the age group ≥ 70, the incidence of O2 and O3 together was 16 per 100,000 person-years. The details in various age and gender groups are presented in Table 1. However, in the age group ≥ 70, the annual incidences of O2 and O3 fractures together almost tripled from 7.4 per 100,000 person-years in 2002 to 22.1 per 100,000 person-years in 2014 (Figure 3). There was a positive trend over the observed years for the incidence of all odontoid fractures (O2 and O3) of patients ≥ 70 years of age (*r* = 0.71, *p* < 0.01) (Figure 3). No correlation was found for other C2 fracture subtypes.

### 4.3. Treatment

**Figure 4:**
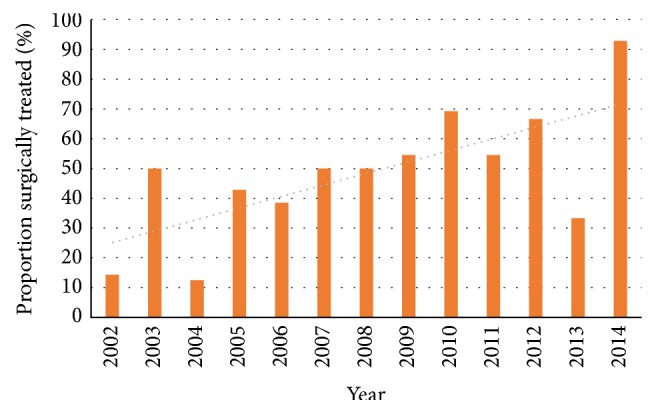
Proportion of patients with odontoid fracture type 2 treated surgically 2002–2014.

In total 40% of all C2 fractures were treated surgically. Of O2 fractures 53% received surgical treatment, 63% in the age group < 70 years and 50% in in the age group ≥ 70 years, whereas only 19% of the O3 fractures were treated surgically. Of Hangman's fractures 54% were treated surgically. All 11 H2 and 2 of 14 H1 (14%) were operated on. In the subgroup atypical C2 fractures 8% were treated surgically. Three patients in the nonsurgical group were treated with a halo-vest, one O2 and two A. One patient was lost to follow-up and excluded from the treatment analysis due to untraceable relocation. There was no statistical difference between the younger and older age groups (*p* = 0.57) and between gender regarding assignment to surgical treatment (*p* = 0.55). During the observed years, there was a change in treatment strategy observed, concerning O2 fractures, with an increased proportion of patients treated surgically from 2002 to 2014 (*r* = 0.70, *p* < 0.01) (Figure 4). This trend could also be seen in the age group ≥ 70 years (*r* = 0.61, *p* < 0.05). It could not be seen in the other fracture subclasses.

## 5. Discussion

This study presents reliable age specific proportions and incidences of C2 fractures subtypes in a comprehensive regional dataset. Furthermore, the current treatment strategy for C2 fractures has been documented.

### 5.1. C2-Fracture Distribution

Previous publications find among odontoid fractures a distribution of 0–4% of type 1, 60–80% type 2, and 20–39% type 3 according to Anderson and D'Alonzo [10, 14, 17–19]. Subgroup proportions of Hangman's fractures are in 29–72% type 1, 28–69% type 2, and 0–10% type 3 classified according to Effendi et al. [10, 16, 20–22].

#### 5.1.1. Odontoid Fractures

Odontoid fractures type 1 were rare and could only be observed in two younger patients.

Our study found a proportion of odontoid fractures type 2 among odontoid fractures similar to previously published data [10, 14, 17–19]. The proportion of patients with odontoid fracture type 2 increased with age (Figure 1). Over the investigated years there was change in odontoid fracture subgroup distribution. In the older age group, we could see a reduced proportion of odontoid fractures type 2 in favour of type 3 from 2002 to 2014, although the proportion of odontoid fractures as a total remained the same. The higher rate of the odontoid fracture type 2 proportion in elderly can be explained by the typically osteoporotic nature of this fracture type [23]. Since most Hangman's fractures require a high-energy injury mechanism, they are less common among elderly, indirectly raising the odontoid fracture proportion [22].

The observed annual increase of incidence of odontoid fractures can be caused by a detection bias. The diagnostic procedure applied in this relatively recent cohort includes cervical computed tomography being more sensitive than plain radiographs, which were used in most previously published studies [24]. Even the relative increase of type 3 fractures among odontoid fractures during the last decade could be explained by an improved differentiation between types 2 and 3 in CT scans. Our application of the modified classification by Grauer et al. [15], distinguishing odontoid fracture type 2 and shallow type 3, could have led to a lower proportional estimate of odontoid fractures type 2.

Within the subgroup of odontoid fractures, 30% were odontoid fractures type 3. There was a higher proportion of odontoid fractures type 3 among elderly compared to the younger age group (Figure 1). This could again be caused by the osteoporotic fracture mechanism of odontoid fracture type 3 in elderly [25].

#### 5.1.2. Hangman's Fractures

With only 26 cases in our cohort, the plotted distribution of Hangman's fracture subtypes lacks detail (Figure 2). In this study the Hangman's fracture subgroups were evenly distributed with 54% type 1 and 46% type 2. Exactly the same distribution has been recently published in a cohort of 41 patients with Hangman's fractures, where 54% were type 1 and 46% type 2 according to Al-Mahfoudh et al. [22].

#### 5.1.3. Atypical C2 Fractures

Atypical C2 fractures occurred in 10% of all C2 fractures, 21% of the younger age group, compared to 6% in the age group ≥ 70 years. One earlier study found a proportion of 20% atypical C2 fractures, which is comparable with the younger age group in this study. This group comprises multiple fracture types and fracture mechanisms [9, 26]. The types and proportion of this specific C2-fracture subgroup have not yet been thoroughly investigated. Possibly, most atypical C2 fractures require a high-energy injury mechanism, and are thus more represented within the younger age group [9, 10, 26].

### 5.2. Annual Incidence

From 2002 to 2014 we could observe an increase in the odontoid fractures type 2 incidence, from 7 to 16 per 100,000 person-years. The same trend has been shown in some earlier studies [12, 27, 28]. While several authors blame the ageing population for this development [12, 29], the proportion of the elderly population did not increase in Uppsala and Malmö from 2002 to 2014. The reason for this specific population development is most likely the urban nature of the included municipalities and the migration of the younger population towards cities in Sweden [30].

Elderly patients have a higher level of activity nowadays, with a higher risk of fractures. In Sweden the proportion of institutionalised elderly decreases, while more depend on home-care services [31]. Whether there is a correlation between elderly care and risk of C2 fractures is not investigated, yet, and warrants further research.

Beyond that, improved diagnostic protocols, that is, the standardised use of CT scans in cervical injury treatment algorithms, decreased the possibility of missed fractures, which artificially increases the incidence of fractures.

### 5.3. Treatment

Regarding the treatment of odontoid fracture type 1 most authors recommend nonsurgical treatment [9, 10]. Treatment trends for odontoid fractures type 1 are impossible to evaluate in our study, as there were only two patients with odontoid type 1 fracture.

In the absence of high-level evidence, the treatment rationale of odontoid fractures type II of the elderly has been a matter of debate. In the US there is a trend towards surgical management of these fractures [1, 12, 23, 27, 32–34]. In this study a trend towards surgical management was found, as well. This trend was seen both for the younger population and for the elderly. These results cannot be generalised for the rest of Sweden, as the nonavailability of cervical spinal expertise in rural areas combined with a resilience of patients and doctors to a long-distance referral, a common anaesthesiologist-driven fear of complications in elderly patients [35], and the seemingly obvious economic advantage of cervical orthoses over costly surgical procedures [36] motivate surgeons to use nonsurgical treatment.

With regard to the treatment of odontoid type 3 fractures there is a consensus on nonsurgical treatment, such as a collar or halo-vest [37, 38]. The treatment rationale of odontoid fractures type 3 in our study followed these recommendations.

Regarding treatment of Hangman's fracture type 1, nonsurgical treatment with a rigid collar was dominating. Hangman's fractures type 1 are a domain of nonsurgical treatment, while for types 2 and 3 fractures there is a consensus on surgical treatment depending on the degree of displacement [21, 39]. In this study all 12 Hangman's fractures type 2 underwent surgery.

There are no recommendations on the treatment of the atypical C2 fracture, since it summarises multiple unclassifiable fracture types. In this study, most of the atypical C2-fractures were treated nonsurgically with an external brace, and two patients were treated with a halo-vest.

### 5.4. Limitations of This Study

One major limitation of retrospective epidemiological studies is lost cases due to incomplete registration or incorrect coding. In theory, patients treated entirely outside of their county, and patients not seeking medical assistance for their neck injury, could be missing in our dataset. The availability of a national patient registry with >90% coverage of fracture cases allows proper identification of most C2 fracture cases in the investigated regions [40].

During our radiographic analysis and patient history screening, no cases had to be excluded as false-positive, confirming the quality of diagnosis coding. While plain radiographs miss about 23% of cervical spinal injuries [41], CT protocols, as used in our department, have near 100% sensitivity for cervical injuries [42]. We therefore assume that very few injuries were missed in our cohort.

About 5% of patients with cervical spine injuries primarily do not seek any medical assistance for their neck injury [41]. This relatively small proportion should not have affected the distribution or the incidence of C2 fractures in our study population, since most of these patients will eventually be referred to radiological diagnostics, and will then appear in one of the spine surgical departments. No patient in our dataset was referred with delay; therefore we consider the number of missed spinal injuries negligible.

Since our cohort was treated at two highly specialised level-one trauma centres, a selection bias could be assumed, since elderly patients are less likely to receive treatment at level-one trauma centres [43]. In Sweden, in case of cervical injuries, the responsible level-one trauma centre is always contacted, and the cervical injury thus registered with this centre. Therefore, this selection bias will not lead to missed cases in the elderly population.

Beyond that, the regions of Uppsala and Malmö are highly urbanized. The population of 14% of Swedes living in the rural countryside was therefore not well-represented in our cohort [30]. The analysis of treatment differences due to accessibility requires a national cohort study design.

With this study the previously published distribution of C2 fracture subgroups has been validated, and new values for subgroup proportions presented for regions in Sweden. These values allow further population-based research where subgroup proportions have to be estimated from ICD-10 codes. The proportion of surgically treated odontoid fractures type 2 increased during the last decade in Uppsala and Malmö, which follows the recent development of recommendations. National population-based studies could improve the current evidence and strengthen treatment guidelines.

## Figures and Tables

**Table 1:** Population-at-risk and annual incidence (per 100,000 person-years) of C2 fractures divided into odontoid, Hangman's, and atypical fractures during the years 2002 to 2014.

Age and gender	Population-at-risk	Annual incidence (per 100,000 person-years) 2002–2014			
		Odontoid fractures	Hangman's fractures	Atypical fractures	Total
Men <70	201,402	1.0	0.4	0.5	1.9
Women <70	201,444	0.5	0.2	0.03	0.8
Men ≥70	28,713	15.8	0.8	1.3	17.9
Women ≥70	41,382	16.0	1.1	0.7	17.8
Total	472,941	3.0	0.4	0.4	3.8
